# On the Development of a Release Mechanism for a Split Hopkinson Tension and Compression Bar

**DOI:** 10.3390/ma14247609

**Published:** 2021-12-10

**Authors:** Georg Baumann, Dominik Niederkofler, Christian Ellersdorfer, Florian Feist

**Affiliations:** Faculty of Mechanical Engineering and Economic Sciences, Vehicle Safety Institute, Graz University of Technology, 8010 Graz, Austria; dominik.niederkofler@tugraz.at (D.N.); christian.ellersdorfer@tugraz.at (C.E.); florian.feist@tugraz.at (F.F.)

**Keywords:** brittle failure, release mechanism, Split Hopkinson bar, trigger duration

## Abstract

Split Hopkinson bars are used for the dynamic mechanical characterisation of materials under high strain rates. Many of these test benches are designed in such a way that they can either be used for compressive or tensile loading. The goal of the present work is to develop a release mechanism for an elastically pre-stressed Split Hopkinson bar that can be universally used for tensile or compressive loading. The paper describes the design and dimensioning of the release mechanism, including the brittle failing wear parts from ultra-high strength steel. Additionally, a numerical study on the effect of the time-to-full-release on the pulse-shape and pulse-rising time was conducted. The results of the analytical dimensioning approaches for the release mechanism, including the wear parts, were validated against experimental tests. It can be demonstrated that the designed release concept leads to sufficiently short and reproducible pulse rising times of roughly 0.11 ms to 0.21 ms, depending on the pre-loading level for both the tension and compression wave. According to literature, the usual pulse rising times can range from 0.01 ms to 0.35 ms, which leads to the conclusion that a good average pulse rising time was achieved with the present release system.

## 1. Introduction

In general, engineering disciplines, such as mechanical or civil engineering, strive to design structures or components that are as ductile as possible. The reason for this is to prevent sudden catastrophic, brittle failure. However, there are also applications in which an abrupt, brittle failure of a component is not only tolerated, but it is even desired. One such application is the Split Hopkinson test bench or, more precisely, its release mechanism, which is examined in more detail in the course of this article.

### Split Hopkinson Test Bench

**Figure 1:**
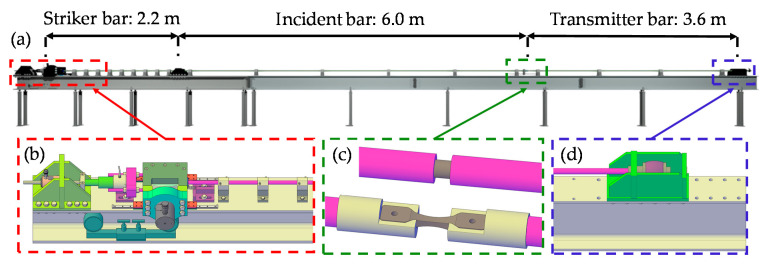
Overview (**a**) and detailed sections of the Split Hopkinson test bench: striker bar with hydraulic pre-tension/compression system and release mechanism to initiate the shock wave (**b**); test specimen (dark brown) positioned between incident and transmitter bar: compression specimen (top) and tensile specimen (bottom) (**c**); damping element in order to absorb excess energy (**d**).

A Split Hopkinson test bench is a facility for the dynamic mechanical characterisation of materials at high strain rates ranging from around 100 s^−1^ up to 10,000 s^−1^ (see Chen and Song [1] and Sunny et al. [2]). Knowledge of the mechanical properties of materials at such high strain rates is essential in the fields of vehicle safety, sports equipment, aerospace, and many other disciplines. The test bench consists of several long bars between which the test specimen is positioned (see Church et al. [3]). An overview of such a system is given in Figure 1.

In the experiment, the striker bar is released towards the incident bar, causing an almost one-dimensional shock wave, or pulse, to form (see Chen and Song [1]). The pulse propagates through the striker bar and the incident bar until it reaches the test specimen and deforms it. Parts of this incoming pulse are reflected back into the incident bar and another part of the pulse is transmitted in the direction of the transmitter bar (see Song and Chen [4]). The pulse propagates at the bar material’s speed of sound. According to Bragov and Lomunov [5], steel or aluminium are usually used for this purpose, resulting in sound speeds of around 5000 m/s. The pulses are recorded with the help of strain gauges attached to the bars (see Meenken [6]). In order to properly track the strain signals sampling rates in the range of at least 100 kHz up to several MHz are required (see Baranowski et al. [7]). Based on the incoming pulse, the reflected pulse, and the transmitted pulse, the stress-strain characteristics of the material sample can be calculated at the respective strain rate.

In practice, there are many different ways to initiate a pulse. One of the most frequently used variants is to propel the striker bar with help of a gas gun (see Nutkani et al. [8]). This variant is mainly used for pressure test stands, although tensile test stands are possible, too. This requires, however, differently designed striker and incident bars, to allow for engagement either in tension or compression.

According to Mancini et al. [9] another option is to build up internal energy in the striker bar by means of elastic pre-stressing and quickly releasing it through a brittle failing wear part. An overview of the already mentioned and further common loading and release systems for Split Hopkins bars, as well as their advantages and disadvantages, is given in Table 1.

The present test rig should be designed in such a way that centric pre-stressing is possible and easy to adapt for the tension and compression case. Furthermore, the pre-loading level in the striker bar should be independent from any material scattering of the wear part. That implies that the wear part doesn’t carry the pre-loading level of the striker bar directly, but indirectly, via a specially designed release mechanism (break load). Apart from this, each individual pre-loading level in the striker bar (tension and compression) should be realised via one single wear part variant. Finally, it has to be investigated if the present mechanical release mechanism (including a brittle failing wear part) is fast enough to allow for a sufficiently short pulse rising time. Contrary to the system presented in Albertini and Montagnani [15] or Albertini et al. [16], the present system should be fitted into an enclosed and compact housing. In order to achieve such a multipurpose test-rig, this work deals with the development of a proper, robust release mechanism. This involves analytical calculations on the wear part and the release mechanism, numerical simulation of the pulse generation as well as the related experimental validation of these functions.

## 2. Materials and Methods

### 2.1. Release Mechanism

In order to hold and quickly release the built-up energy in a reliable and reproducible way, a sophisticated release mechanism is required. A wide variety of approaches were considered and their suitability assessed. Besides clamping bushes, powerful electromagnets, and a kind of scaled-up crossbow mechanism, systems with brittle failing wear parts were considered. Since the former systems proved to be too sluggish in releasing the built-up energy or were not able to hold the required load of 100 kN, the principle of brittle failing wear parts was pursued. The basic idea behind this principle is that the unstable crack growth of a brittle failing material should ensure a sufficiently fast release. (see Mancini et al. [9]) and thus, an almost rectangular pulse.

**Figure 2:**
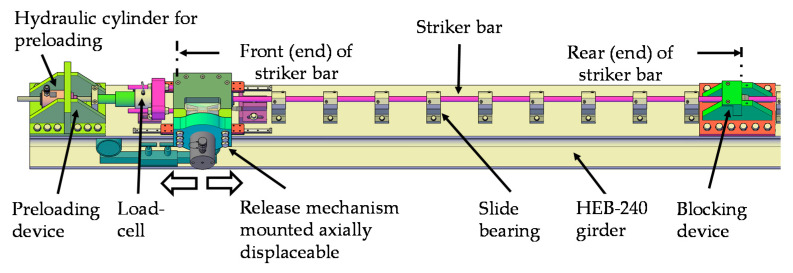
Detailed area of the striker bar with the main elements: pre-loading device, release mechanism, and blocking device.

As mentioned before, an essential requirement of the present test bench was that the pre-loading in the striker bar and, therefore, the amplitude of the generated wave can be set precisely and reproducibly. Therefore, the pre-loading level must be independent from any material variety e.g., of brittle wear parts. For this reason, the force transmission from the hydraulic system to the striker bar was not carried out directly via the wear part, as otherwise the achievable pre-load would vary with its force of failure. Based on these criteria, a concept for a mechanical pre-loading and releasing of the striker bar was developed (see Figure 2).

**Figure 3:**
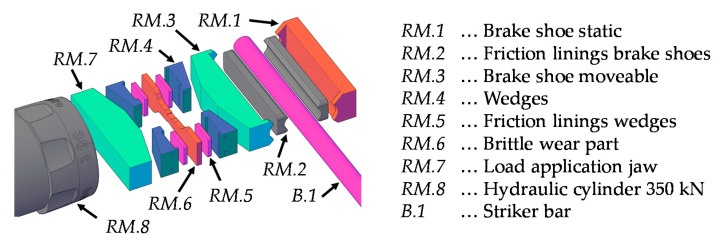
Exploded view of the main components in the release mechanism for clamping, pre-loading, and subsequent abrupt release of the striker bar.

The striker section of the test bench (see Figure 2) consists of the three main elements: pre-loading device, release mechanism, and blocking device. The pre-loading device houses a 125 kN hydraulic cylinder “RCH121” (Enerpac, WI, USA) in order to build up the required energy. Depending on the orientation of the pre-loading device, it can be used in either tension or compression. The striker bar itself (diameter = 20 mm) originates at the release mechanism and ends at the blocking device. While the blocking device represents a non-displaceable abutment, the release mechanism is displaceable along the bar axis. The release mechanism is connected to the pre-loading device via threaded rods and a load cell. First, the front end of the striker bar is clamped in the release mechanism. Then, the release mechanism is displaced. As the rear end of the striker bar is restrained by the blocking device, more and more internal elastic energy builds up. As soon as the required pre-loading level is reached, it can be set free by activating the release mechanism. This leads to the formation of an almost one dimensional shock wave running through the bar system. The release mechanism, which includes the previously mentioned brittle wear part, is shown in Figure 3.

The clamping forces are released through a hydraulic cylinder “HGC 75 S 35” (Holmatro, MD, USA) with a permissible maximum force of 350 kN. The transmission of the clamping force from the hydraulic cylinder (*RM.8*) to the striker bar (*B.1*) consists of several components. First, the load is transferred to the load application jaw (*RM.7*) and further to two pairs of wedges (*RM.4*). The brittle wear part (*RM.6*) is installed between the wedge pairs (*RM.4*) equipped with friction liners (*RM.5*). As a result of the compressive force from the hydraulic cylinder the wear part is subjected to tensile stresses. As long as the wear part is intact, the load is transferred from the wedges (*RM.4*) to the movable brake shoe (*RM.3*). The brake shoes are equipped with friction linings (*RM.2*) to achieve a high pre-loading force for a given clamping force. In between the two frictional linings (*RM.2*) sits the striker bar (*B.1*). On the opposite side of the movable brake shoe (*RM.3*) is the static brake shoe (*RM.1*), which transfers the loads into the housing of the release mechanism. A summary of the main components in the release mechanism is given in Table 2.

**Figure 4:**
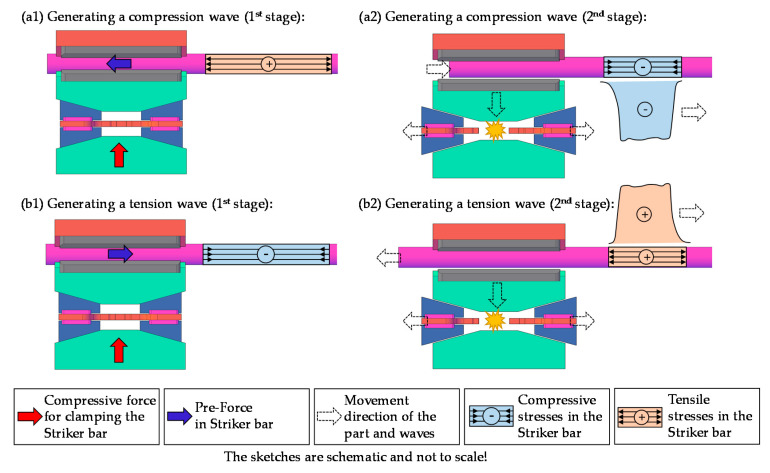
Main stages in the mechanical release mechanism for the generation of compression waves (**a1**) and (**a2**) or tension waves (**b1**) and (**b2**).

In order to build up and maintain a pre-loading force of up to 100 kN in compression or tension without the striker bar slipping, a certain minimum clamping force is required. This clamping force depends on the coefficient of friction of the friction linings (*RM.2*). Depending on the minimum clamping force, the inclination angle of the wedges (*RM.4*), as well as their coefficient of friction, the minimum tensile force that the wear part (*RM.6*) must withstand, can be derived. When the striker bar has been clamped and pre-loaded, it must be released again. In order to abruptly release the striker bar, the load in the hydraulic cylinder (*RM.8*) is increased to the point where the wear part (*RM.6*) fails in a brittle manner. Due to the brittle failure of the wear part (*RM.6*), the wedge pairs (*RM.4*) begin to slide outwards and relieve the pressure on the moveable brake shoe (*RM.3*). This, in combination with the elastic rebound of the involved components, leads to a sudden release of the energy in the striker bar, which causes the pulse to form in the bar system. Note that the generation of a compression wave requires a pre-tension of the striker bar, while a pre-compression leads to the formation of a tension wave. An overview of the compression and tension wave formation and their main stages is given in Figure 4.

In the following sections, the load boundary conditions and the mechanical design of the wear part (*RM.6*), including material selection and geometry determination, are discussed in more detail. Hereby, the wear part should be designed in such a way that each individual pre-loading level in the striker bar (between +/− 100 kN) can be realised by one single wear part variant.

### 2.2. Boundary Conditions and Force Flows in the Release Mechanism

The basis for the design and the dimensioning of the wear part is knowledge of the load boundary conditions. In order to illustrate the complex force flows that occur between the hydraulic cylinder (*RM.8*) and the static brake shoe (*RM.1*), the following sections of the release mechanism are cut free, and the force flows are visualised.

Starting point for the calculation is the required maximum pre-loading force in the striker bar (*F_S_*) of 100 kN. The brake shoes (*RM.1* and *RM.3*) are each equipped with friction linings (*RM.2*). A static friction coefficient (μ_ST_) of 0.48 was determined experimentally between the striker bar (7075 aluminium) and the friction linings. This static friction coefficient results from the experimentally determined static friction angle (ρ*_ST_*) of 25.5° for the mentioned contact partners.

**Figure 5:**
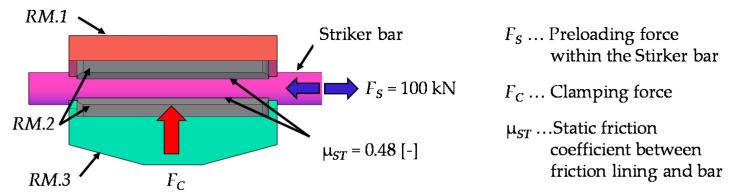
Sketch of the force components between the striker bar and the brake shoes, respectively, with the friction linings.

The static friction angle was determined by means of an adjustable inclined plane. The two contact partners are placed one above the other, with the lower contact partner representing the inclined plane. The angle of the inclined plane is now increased until the rest position of the upper contact partner is disturbed and it begins to slide. The angle of sliding friction is determined in a similar way, but in contrast to before, the upper contact partner is already sliding, and the angle of the inclined plane is reduced until it comes to a standstill. This angle is relevant for the movement of the wedges (*RM.4*) further on. A sketch of the partial area of striker bar clamping in the release mechanism is shown in Figure 5.

Depending on the preload force (*F_S_*) and the static friction coefficient (μ*_ST_*), the minimum clamping force (*F_C,min_*) required to hold the striker bar can be calculated. The maximum possible clamping force (*F_C,max_*) that the hydraulic cylinder (*RM.8*) can apply is 350 kN. Therefore, the clamping force corridor, in which a failure of the wear part must take place, ranges from about 105 kN (*F_C,min_*) to 350 kN (*F_C,max_*). In order to achieve the most reliable release within this corridor, the wear part is dimensioned in such a way that failure would most likely occur in the middle between these two limits. This consideration is based on the assumption of a normally distributed and, thus, symmetrical dispersion of the breaking load of the wear part as well as of further influencing factors, such as friction between the moving components. Therefore, the mean clamping force (*F_C,mean_*, hereafter referred to as *F_C_* for short) is 227.5 kN.

**Figure 6:**
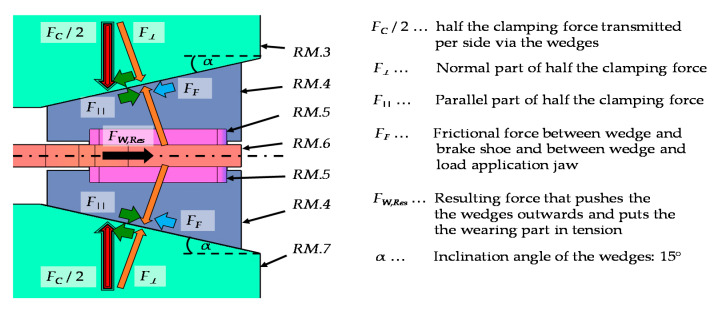
Sketch of the force components between the wedges, the moveable brake shoe, the load application jaw, as well as the resulting force in the wear part for the right half of the system.

Figure 6 below shows the force components between the movable brake shoe (*RM.3*), the wedges (*RM.4*), the wear part (*RM.6*), and the load application shoe (*RM.7*) for the right half of the system. Here, the wear part (*RM.6*) is clamped between the two wedges (*RM.4*). In order to achieve the best possible power transmission to the wear part (*RM.6*), the wedges (*RM.4*) are equipped with friction linings (*RM.5*).

The normal part (*F_⊥_*), as well as the parallel part (*F_||_*), of half the clamping force (*F_C_*/2) result to 109.9 kN and 29.4 kN. The moveable brake shoe (*RM.3*), the load application jaw (*RM.7*), as well as the wedges (*RM.4*) are each made of hardened steel (CK45) and are grinded in their contact areas. A thin layer of graphite grease is applied to lubricate the friction surfaces. The experimentally determined sliding friction angle (ρ*_SL_*) is 13°, which corresponds to a sliding friction coefficient (μ*_SL_*) of around 0.23. Since the friction surfaces are subject to high mechanical loads, and the lubrication cannot be kept completely constant from test to test, this coefficient of friction is subject to certain dispersion. The friction force (*F_F_*) which is calculated by the normal force part (*F_⊥_*) and the sliding friction coefficient (μ*_SL_*) results in 25.4 kN.

The resulting force (*F_W,Res_*) in the wear part consists of the cosinus components from the counteracting forces (*F_F_*) and (*F_||_*). The static friction coefficient (μ*_ST_*) between the wedge friction linings (*RM.5*) and the wear part (*RM.4*) was determined analogously to the method of the inclined plane already presented. With a value of 0.51, it is significantly larger than the coefficient of sliding friction (μ*_SL_*) of 0.23 between the lubricated wedges (*RM.4*) and jaws (*RM.3* and *RM.7*). Therefore, it can be assumed that the wedges (*RM.4*) and the wear part (*RM.6*) move outwards synchronously and without slip. This leads to a resulting tensile force in the wear part (*F_W,Res_*) of 7.9 kN, which is the basis for the further dimensioning. A summary of all forces, which are acting on the components of the release mechanism, are given in Table 3. Hereby, the mean force level, which is most likely to occur during the release, as well as the maximum forces which can be applied, are listed.

### 2.3. Material Selection

Based on the previously derived boundary conditions in the release mechanism, as well as the general test bench requirements, the following criteria arise for the material selection of the wear part:

1. A low toughness to ensure brittle and abrupt failure.
2. A sufficient resistance against a tensile load of 7.9 kN, acting in axial-direction, as well as against the compressive clamping forces of up to 350 kN acting in thickness-direction.
3. A low scattering of the mechanical properties of the wear part in order to achieve a failure in the specified force corridor. Besides the wear part itself, there must also be a buffer for the scattering of the coefficient of sliding friction (μ*_SL_*) between the wedges (*RM.4*) and the surrounding components (*RM.3* and *RM.7*).
4. A material and production price which is economically justifiable, since a high number of wear parts is required, which, in turn, affects the test costs.

According to these criteria, suitable materials were pre-selected by using the CES Edu Pack 2018 material database. After applying appropriate search filters, only a small selection of high-strength and ultra-high-strength steels remained. Among them were the products M340 [17] and N680 [18], which were available from a local supplier. For the heat treatment vacuum, hardening at 980 °C, followed by nitrogen quenching and tempering, twice, at 540 °C was foreseen. According to the supplier, guide values for the ultimate tensile strength (*f_u_*), after quenching and tempering, are given to 1500 MPa.

Although an omission of the tempering process would probably lead to a more brittle material behaviour, no certain guide values are known for this case. Therefore, the intended heat treatment was used for this steel. Initially, the focus was on M340 for the production of bolt-shaped wear parts. However, as a flat sample is significantly cheaper to produce in large quantities, further design steps were carried out on the basis of the N680 with a sheet thickness of 5.5 mm (cf. Figure 3). Once the material was selected, the geometry of the wear part was identified.

### 2.4. Geometry Definition

**Figure 7:**
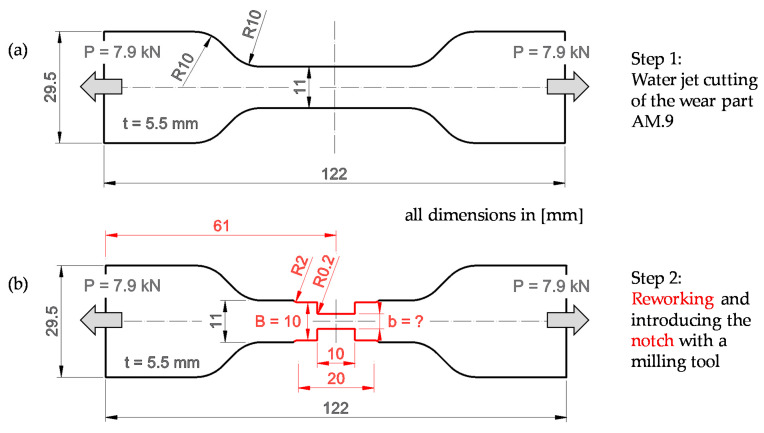
Contour of the wear part after water jet cutting (**a**) and after milling (**b**), as well as the geometric parameters needed for the nominal stress concept and the loading values.

The overall dimensions of the wear part, such as length and width, are given by the space in the housing of the release mechanism. The length (*l* = 122 mm) corresponds to the distance between the outer edges of the two pairs of wedges (*RM.4*) seen in Figure 4(a1,b1). As the wear part (*RM.6*) is clamped upright between the wedges (*RM.4*), the width (*w* = 29.5 mm) corresponds to the thickness of the wedges. There are two main steps in shaping the wear part. The first step is generating the basic shape by means of water jet cutting. In a second step, the central 20 mm in the tapered area of the wear part is reworked, including a 10 mm long notch, by using a milling tool (see Figure 7a,b). An overview of the geometric parameters and the acting forces, which are needed for the dimensioning of the wear part are also given.

Reworking to a width of *B* = 10 mm is necessary because precisely defined reference edges are required for exact notch insertion. The radius at the transition between the waterjet-cut edge and the machined edge is 2 mm, whereas the radius at the base of the notch is 0.2 mm. A notch with a 0.2 mm radius can be reproduced sufficiently well with standard milling tools and should lead to a sufficiently high stress concentration. The shaping by means of waterjet cutting and milling was carried out in the soft-annealed material state. The heat treatment process described in Section 2.3 follows the shaping. The determination of the required notch depth, or residual width of the notched area (*b*), will be discussed in the following sections.

### 2.5. Calculation of the Notch Depth Using the Nominal Stress Concept

In the nominal stress concept, the determination of the notch depth basically corresponds to a classical strength verification. In order to consider the effect of the notch, stress concentration factors are introduced. This method assumes a quasi-brittle material behaviour. A pronounced plastic behaviour would lead to a reduction in the stress concentrations in the base of the notch and, consequently, to a more uniform stress distribution in the remaining cross-section. According to Steinhilper and Sauer [19], the determination of the stress concentration factor (α*_K_*) for a double edge notched tensile specimen results from Equation (1). A sketch of the essential geometric parameters and loading values, according to the formula, is shown in Figure 7b.

(1) αK=1+1A(B−b2×r)k + E×(1+b2×r(b2×r)3)l+ GbB×(B−b2×r)m

The width (*B*) corresponds to the unnotched area of the sample, which is 10 mm. The dimension (*b*) is the residual within the notched area and, thus, results from the width (*B*) minus twice the notch depth. The sharpness of the notch base is characterised by its radius (*r*) of 0.2 mm. The unitless parameters—*A* = 0.10; *E* = 0.70; *G* = 0.13; *k* = 1.00; *l* = 2.00, and *m* = 1.25—are given by the wear part’s shape and the type of loading. According to Steinhilper and Sauer [19], the maximum stress (*σ_max_*) in the notch base corresponds to the nominal stress scaled with the stress concentration factor (α*_K_*) (see Equation (2)).

(2) $$\sigma_{max}=\frac{P}{b\times t}\times \alpha_{K}=\;\sigma_{nenn}\times \alpha_{K}$$

**Figure 8:**
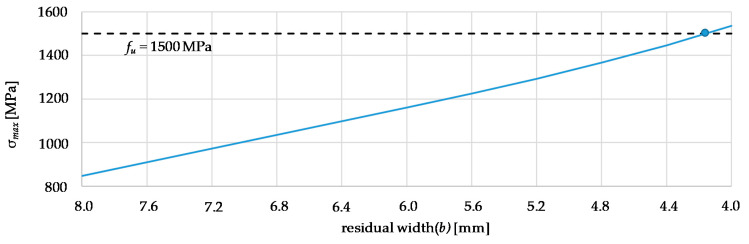
Variation study to determine the influence of the residual width (*b*) on the maximum stress (*σ_max_*).

The nominal stress is calculated from the acting force (*P*), which is divided by the residual width (*b*) and the thickness (*t*). The stress concentration factor (α*_K_*) and the maximum stress (*σ_max_*) were evaluated for successively decreasing residual widths (see Figure 8).

In order to achieve a failure of the wear part, the maximum stress (σ*_max_*) must exceed its ultimate tensile strength (*f_u_*), which is around 1500 MPa for the quenched and tempered state. According to the nominal stress concept, this results in a residual width (*b*) of 4.2 mm.

### 2.6. Numerical Study on the Influence of the Trigger Duration in the Release Mechanism

In addition to the determination of the suitable breaking load of the wear part, another essential aspect is a sufficiently fast release of the entire mechanism. The generated initial shock wave, which represents the load pulse on a specimen, should be close to a rectangular shape (see Mancini et al. [9]). Larger deviations from an idealised rectangular shape can lead to inaccuracies in the determination of the stress-strain characteristics but also to deviations from the targeted strain rate for testing. In order to estimate the influence of the trigger duration on the quality of the shock wave, a simplified model of the test bench was established. The model consists of the three long and slender bars (striker, incident, and transmitter) with a diameter of 20 mm. Furthermore, a cylindrical compression specimen (diameter = height = 12 mm), as well as a dog bone shaped tensile specimen (length = 75 mm and overall width = 15 mm), which can be seen in Figure 1c, were modelled. The compression specimen was positioned between the faces of the incident and the transmitter bar while, for the tensile specimen, two additional sleeves were foreseen in order to clamp it. A EN AW-7075 aluminium alloy was assumed for the bars and the sleeves, while the specimens were assumed from a EN AW-1050 aluminium alloy. Both the bars and the specimen were meshed using 8-node hexahedrons with an element size of around 1.0 mm. For the simulation of the bars and specimens the material model “*Mat_Piecewise_Linear_Plasticity (*Mat_024)” [20] was used within the explicit solver LS-Dyna R13 SMP.

The pre-loading of the striker bar was applied by a forced node displacement. Two different force levels (30 kN and 74 kN) were simulated. Additionally, four different unloading processes were also considered, where the pre-strain forces were assumed to decrease linearly with time. In a first run, an idealised, completely delay-free release situation was calculated. For the next runs times-to-full-releases of 0.25 ms, 0.50 ms, and 0.75 ms were considered.

### 2.7. Experimental Validation of the Analytical Calculations and Numerical Simulation Results

In order to check the suitability of the analytically dimensioned wear part, as well as to guarantee a sufficiently rapid activation of the release mechanism, validation experiments were subsequently carried out in the test stand. Within the validation, the following functionalities were checked, and the following properties were analysed:

1. Ensuring a reliable transfer of the required minimum clamping force (*F_C,min_*) of 105 kN to the striker bar.
2. Ensuring a reliable activation of the release mechanism within the specified clamping force corridor of 105 kN to 350 kN.
3. Ensuring a sufficiently fast release of the stored elastic energy within the striker bar.
4. Observation of the clamping force build up until the failure of the wear part is reached.
5. Analyzation of the wear part and its fracture surfaces after the test has been carried out.

**Figure 9:**
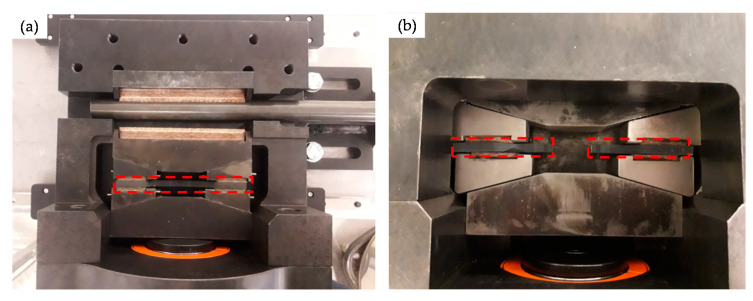
Overview photo of the installed wear part (with open housing cover) before a test (**a**) and failed wear part (with closed housing cover) after a test (**b**).

Several wear parts were successively positioned in the release mechanism and stressed according to the test procedure described in Section 2.1. Figure 9 shows photos of the release mechanism with the wear part installed, both before and after the test. The position of the wear part and the separated wear part halves is highlighted by red dashed frames.

The clamping force was monitored optically via the hydraulic pressure gauge. Therefore, the determination of the breaking force was only possible in an approximate manner (±5 kN). To evaluate the quality of the mechanical release, the initial pulse in the incident bar was measured using strain gauges “1-LY13-1.5/120“ (HBM, Vienna, Austria). Using the amplifier “Dewe3-M4” in combination with a “Trion-1820-Multi-4-D-Card” (both Dewetron, Grambach, Austria) a sampling rate of 2 MHz was achieved. The pre-load in the striker bar was controlled with a loadcell “K-K12/N520-G21” with a capacity of 200 kN (Lorenz Messtechnik GmbH, Alfdorf, Germany). An acceleration sensor “Model 1201” with a capacity of up to 1000 g (TE connectivity, Hampton, VA, USA) is applied on the housing of the release mechanism in order to trigger the strain gauges.

## 3. Results and Discussions

**Figure 10:**
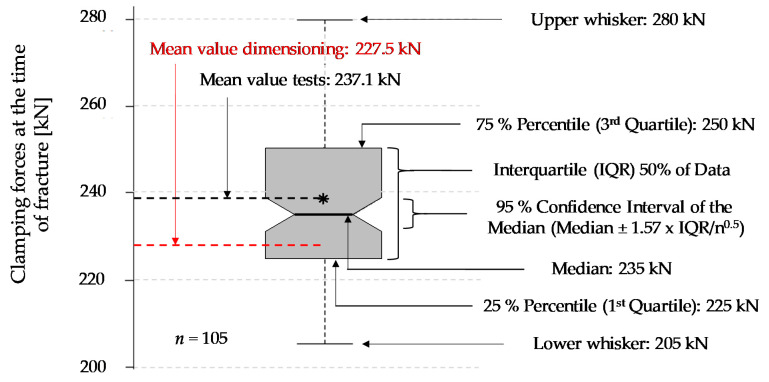
Boxplot of the experimentally determined clamping forces at the time of rupture of the wear parts and comparison with the design clamping force.

**Figure 11:**
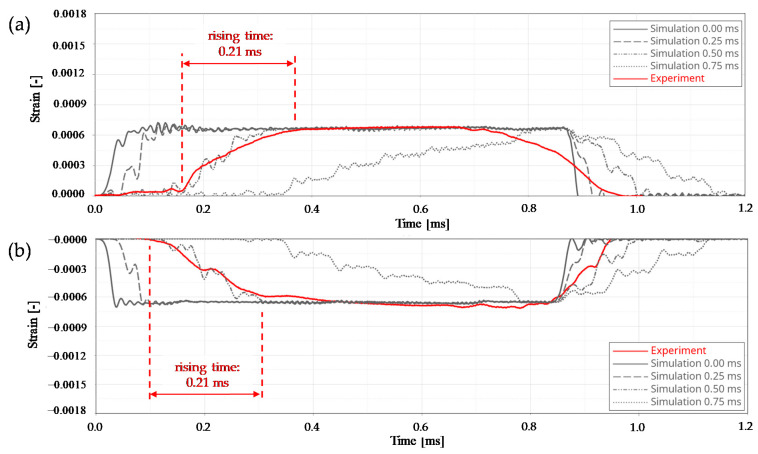
Comparison of the experimental and numerical initial incoming pulses in the incident bar, at a pre-load of 30 kN, for a tensile wave (**a**) and a compression wave (**b**).

**Figure 12:**
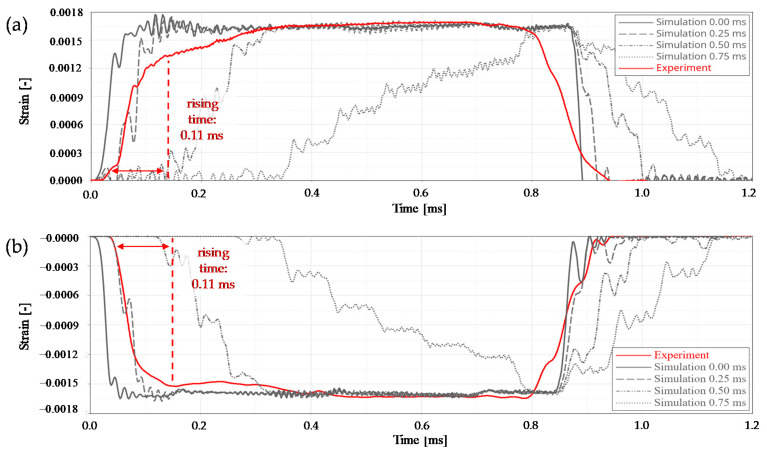
Comparison of the experimental and numerical initial incoming pulses in the incident bar, at a pre-load of 74 kN, for a tensile wave (**a**) and a compression wave (**b**).

**Figure 13:**
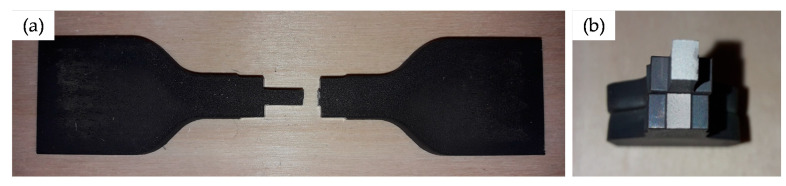
Exemplary photo of the failed wear part after removal (**a**) and detailed photo of the fracture surface (**b**).

Based on the conducted calculations and experimental validation tests the following results were obtained:

1. All wear parts transferred the required minimum clamping force (*F_C,min_*) of 105 kN to the striker bar in a reliable manner.
2. The failure of all wear parts tested (*n* = 105) occurred within the specified clamping force corridor. The obtained failure loads ranged from 205 kN to 280 kN with a mean value of 237.1 kN and a coefficient of variation of 6.1%. In comparison, the design clamping force (*F_C_*) for the nominal stress concept was 227.5 kN. The results are shown as boxplot in Figure 10.
3. The evaluation of the release duration was based on the achievable incoming pulse shapes in the incident bar. Four different pre-loading scenarios were distinguished. On the one hand, a moderate pre-loading of 30 kN (see Figure 11) and on the other hand, a relatively high pre-loading of 74 kN (see Figure 12). Both pre-loading levels were simulated and tested for the compression as well as the tensile scenario. In case of the 30 kN pre-loading levels, the experimentally determined pulses most closely resembled a simulated time-to-full-release of 0.50 ms. This leads to a rising time of roughly 0.21 ms for the tensile and compression pulse, which is less than half the trigger duration. The pulse shape still sufficiently approximates an ideal rectangular pulse, even though the edges are clearly inclined and rounded.
  For the variants with 74 kN pre-load, the experimental pulses were best comparable with the simulated time-to-full-release of 0.25 ms. This results in a nice square pulse with much steeper edges and less rounding. The rising time is roughly half the time-to-full-release, namely 0.11 ms. At this point, the strain plateau was almost reached, and there is only a slight decrease up to the maximum strain level. The highest occurring strains are almost identical for all simulated and the experimental pulses.
  For the given release mechanism, the rising times are in the order of 0.11 ms to 0.21 ms. Mancini et al. [9] mentions rising times of conventional Split Hopkinson bars that can be as short as 0.05 ms. The mechanical release of their study achieved rising times between 0.06 ms and 0.12 ms, depending on the heat treatment condition of the wear part. An overview study on Torsional Split Hopkinson bars, conducted by Yu et al. [14], showed that pulse rising times can be in a relatively wide range, starting from less than 0.01 ms up to 0.35 ms, depending on the release system. With regard to the initial incoming pulse shape, it is not possible to draw a sharp line as to when this is no longer suitable for dynamic characterisations, as this is also strongly dependent on the test specimen. In general, it can be stated that the brittle failing wear parts provide sufficiently abrupt trigger durations for most measurement tasks by contributing to the generation of an approximate rectangular pulse. Due to the reason that this work deals with the development and evaluation of the release mechanism and not with the whole characterisation process, only the incoming pulses were shown. However, an example of an entire material characterisation task, using the present test bench, is given in Werling et al. [21].
4. The clamping force was monotonically increasing over the entire loading cycle and reached its maximum at the time of sudden failure. This suggests that there is no or hardly any necking in the wear part and, therefore, it also suggests a rather brittle material behaviour.
5. The observation described in point 4 is also reflected in the exemplary wear part sample (Figure 13). The notch base was measured before and after the test with a digital sliding gauge (resolution ± 0.01 mm). No significant difference was found between the original and the final cross-section in the fracture area. This also indicates that there is no or hardly any necking.

## 4. Conclusions

Within this work, it was shown that it is possible to design a mechanical release mechanism, with brittle failing wear parts, for a Split Hopkinson tension and compression bar. Flat double notched tensile specimens made from an ultra-high-strength N680 steel and relatively sharp notches were used as wear parts. The wear part was able to meet the requirements of the test bench, with regards to abrupt failure, in the specified load corridor and without any influence on the pre-loading level in the striker bar. Apart from this, all required pre-loading levels between +/− 100 kN could be achieved with one single wear part geometry. It was further found that the time-to-full-release is, roughly, in the range of 0.25 ms to 0.50 ms, depending on the pre-loading level in the striker bar. The higher the pre-loading level, the shorter the time-to-full-release in the experiment and the better the agreement with an idealised, rectangular incoming pulse in the incident bar. The observed rising times were approximately half the time-to-full-release, i.e., in the range from 0.11 ms to 0.21 ms, for both the tension and compression wave. It can be concluded that the presented release mechanism is capable of generating proper load pulses, which are a good average when comparing pulse rising times from literature, ranging from 0.01 ms to 0.35 ms.

## Figures and Tables

**Table 1:** Overview of common loading and release systems for Split Hopkins bars.

System		Advantages	Disadvantages	Ref.
Direct impact loading	via pendulum	- Relatively short pulse rising time	- Conversion from tension to compression mode is rather elaborate	Li et al. [10],
		- Relatively easy to reproduce certain pulse levels	- Rather bulky design	Leung and Yu [11]
	via gas gun	- Relatively short pulse rising time	- Difficult to reproduce certain pulse levels	Nutkani et al. [8],
				Baranowski et al. [12],
		- Relatively compact design	- Conversion from tension to compression mode is rather elaborate	Acosta [13]
Explosive		- Extremely short pulse rising time	- Difficult to reproduce certain pulse levels	Chen and Song [1],
Loading		- Relatively compact design	- Conversion from tension to compression mode is rather elaborate	Yu et al. [14] *
Electromagnetic loading		- Relatively compact design	- Relatively long pulse rising time	Yu et al. [14] *
Flywheel		- Relatively compact design	- Relatively long pulse rising time	Yu et al. [14] *
Pre-loaded striker bar	Wear part is carrying pre-load	- Relatively short pulse rising time	- Scattering of the wear part influences the preloading level of the striker bar	Mancini et al. [9],
		- Easy to combine tension and compression loading in a single test bench	- Various different wear parts are needed to realize individual pre-loading levels	
	Wear part is carrying break load	- Easy to combine tension and compression loading in a single test bench	- Due to the inertia of the parts in the release mechanism a longer pulse rising time is expected	Albertini and Montagnani [15],
		- Pre-loading level in the striker bar is not influenced by the material scattering of the wear part		Albertini et al. [16],
		- One wear part variant is enough to realize every pre-loading level		Present study

This table is meant as an overview and should not be understood as a comprehensive list. * Torsional Split Hopkinson bar.

**Table 2:** Summary of the main components in the release mechanism, as well as the used materials and their purpose.

Part Number	Material	Purpose
*B.1*	EN AW 7075 Al	Storage of elastic energy and shock wave formation
*RM.1*	CK 45 steel	Clamping of the striker bar and load distribution to the housing
*RM.2*	custom made composite	Increasing the friction coefficient between striker bar and clamping mechanism
*RM.3*	CK 45 steel	Clamping and load distribution to the striker bar
*RM.4*	CK 45 steel	Load transmission and fixation of the wear part
*RM.5*	custom made composite	Increasing the friction coefficient between wedges and wear part
*RM.6*	N 680 steel	Ensuring the transmission of a minimum clamping force and a fast release of the energy in the striker bar
*RM.7*	CK 45 steel	Load distribution from the Hydraulic cylinder to the rest of the clamping mechanism
*RM.8*	-	Generation of the necessary clamping force

**Table 3:** Summary of the mean and maximum forces, which are acting on the components during release.

Part Number	Mean Forces at Release	Maximum Forces at Release
*B.1*	-	+/− 100 kN
*RM.1*	−227.5 kN	−350 kN
*RM.2*	−227.5 kN	−350 kN
*RM.3*	−227.5 kN	−350 kN
*RM.4*	−113.8 kN (per side)	−175 kN (per side)
*RM.5*	−113.8 kN (per side)	−175 kN (per side)
*RM.6*	+7.9 kN	+12.2 kN
*RM.7*	−227.5 kN	−350 kN
*RM.8*	−227.5 kN	−350 kN

+ … tensile loading, − … compression loading.

## Data Availability

All the data is available within the manuscript.

## References

[1] Chen W., Song B. (2011). Split Hopkinson (Kolsky) Bar, Design, Testing and Applications.

[2] Sunny G., Yuan F., Prakash V., Lewandowski J. (2008). Design of inserts for split Hopkinson pressure bar testing of low strain-to-failure materials. Soc. Exp. Mech..

[3] Church P., Cornish R., Cullis I., Gould P., Lewtas I. (2014). Using the split Hopkinson pressure bar to validate material models. Philos. Trans. A Math. Phys. Eng. Sci..

[4] Song B., Chen W. (2005). Split Hopkinson pressure bar techniques for characterizing soft materials. Lat. Am. J. Solids Struct..

[5] Bragov A.M., Lomunov A.K. (1995). Methodological aspects of studying dynamic material properties using the Kolsky method. Int. J. Impact Eng..

[6] Meenken T. (2007). Charakterisierung Niederimpedanter Werkstoffe unter Dynamischen Lasten.

[7] Baranowski P., Gieleta R., Malachowski J., Damaziak K., Mazurkiewicz L. (2014). Split Hopkinson pressure bar impulse experimental measurement with numerical validation. Metrol. Meas. Syst..

[8] Nutkani M.B., Abid M., Pasha R.A., Dar U.A. (2018). Indigenous design and development of split Hopkinson pressure bar (SHPB) test setup for characterization of materials at high strain rates. IOP Conf. Ser. Mater. Sci. Eng..

[9] Mancini E., Sasso M., Rossi M., Chiappini G., Newaz G., Amodio D. (2015). Design of an innovative system for wave generation in direct tension–compression split Hopkinson bar. J. Dyn. Behav. Mater..

[10] Li S.H., Zhu W.C., Niu L.L., Dai F. (2017). Constant strain rate uniaxial compression of green sandstone during SHPB tests driven by pendulum hammer. Hindawi Shock. Vib..

[11] Leung M.Y., Yu T.-X. (2008). Dynamic characterization of micro-scaled samples using the Hopkinson tensile bar technique. J. Strain Anal. Eng. Des..

[12] Baranowski P., Malachowski J., Gieleta R., Damaziak K., Mazurkiewicz L., Kolodziejczyk D. (2013). Numerical study for determination of pulse shaping design variables in SHPB apparatus. Bull. Pol. Acad. Sci. Tech. Sci..

[13] Acosta J.F. (2012). Numerical and Experimental Studies on the Use of Split Hopkinson Pressure Bar for High Strain Rate Tension Testing. Ph.D. Thesis.

[14] Yu X., Chen L., Fang Q., Jiang X., Zhou Y. (2018). A Review of the torsional split Hopkinson bar. Adv. Civ. Eng..

[15] Albertini C., Montagnani M. (1994). Study of the true tensile stress-strain diagram of plain concrete with real size aggregate; need for and design of a large Hopkinson bar bundle. J. Phys. IV Proc. EDP Sci..

[16] Albertini C., Cadoni E., Solomos G. (2014). Advances in the Hopkinson bar testing of irradiated/non-irradiated nuclear materials and large specimens. Phil. Trans. R. Soc. A.

[17] Voestalpine BÖHLER Edelstahl GmbH & Co KG Plastic Mould Steel, Böhler M340 Isoplast. https://www.bohler-edelstahl.com/en/products/m340/.

[18] Voestalpine BÖHLER Edelstahl GmbH & Co KG: Knife Steels. https://www.bohler-bleche.com/en/products/n680/.

[19] Steinhilper W., Sauer B. (2006). Konstruktionselemente des Maschinenbaus 1, Grundlagen der Berechnung und Gestaltung von Maschinenelementen.

[20] Livermore Software Technology (LST) (2021). An Ansys Company. LS-Dyna R13 Keyword User’s Manual Volume II Material Models. https://www.dynasupport.com/manuals/ls-dyna-manuals/ls-dyna_manual_volume_ii_r13.pdf/view.

[21] Werling T., Baumann G., Feist F., Sinz W., Ellersdorfer C. (2021). On the dynamic electro-mechanical failure behaviour of automotive high-voltage busbars using a split Hopkinson pressure bar. Materials.

